# A “Dupla Dinâmica”: O Novo Manejo no Tratamento Medicamentoso da Insuficiência Cardíaca com Fração de Ejeção Levemente Reduzida ou Preservada

**DOI:** 10.36660/abc.20240676

**Published:** 2025-05-08

**Authors:** Plínio José Whitaker Wolf, Edileide Barros Correia, João Manoel Rossi, Marco Aurelio Finger, Carolina Casadei Santos, Marcos de Oliveira Vasconcellos, Larissa Ventura Ribeiro Bruscky, Ana Cristina de Souza Murta, Yoná Afonso Francisco, Fabiano Castro Albrecht, Juliana Jangelavicin Barbosa, Eduardo Mikio Sassaki, Bruno Noshang Blaas, Bianca Fernandes Távora Arruda, Fernanda de Brito Fortuna, Victor Bemfica de Mello Mattos

**Affiliations:** 1 Instituto Dante Pazzanese de Cardiologia São Paulo SP Brasil Instituto Dante Pazzanese de Cardiologia, São Paulo, SP – Brasil; 2 Centro de Referência e Treinamento DST/Aids-SP São Paulo SP Brasil Centro de Referência e Treinamento DST/Aids-SP, São Paulo, SP – Brasil; 3 Escola Paulista de Medicina Universidade Federal de São Paulo São Paulo SP Brasil Escola Paulista de Medicina da Universidade Federal de São Paulo, São Paulo, SP – Brasil

**Keywords:** Insuficiência Cardíaca, Inibidores do Transportador 2 de Sódio-Glicose, Antagonistas de Receptores de Mineralocorticoides

## Abstract

O “Quarteto Fantástico”, termo criado em 2021 para se referir aos quatro pilares medicamentosos no tratamento da insuficiência cardíaca com fração de ejeção reduzida (betabloqueadores, inibidores do sistema renina-angiotensina e neprilisina, antagonistas do receptor de mineralocorticoide e inibidores do cotransportador de sódio e glicose II, ou iSGLT2), apresenta excelente desempenho na redução de morbimortalidade nesse cenário. No entanto, no caso da insuficiência cardíaca com fração de ejeção levemente reduzida ou preservada, os mesmos benefícios não foram observados com esse tratamento em conjunto, restando, por muitos anos, apenas o uso de diuréticos e o controle de comorbidades como manejo recomendado nesse contexto. Contudo, recentemente, novas opções terapêuticas demonstraram eficácia na redução dos desfechos cardiovasculares nesse grupo específico da insuficiência cardíaca com fração de ejeção levemente reduzida ou preservada: a “Dupla Dinâmica” composta pelos iSGLT2 e Finerenona, além despontamento da semaglutida como tratamento “coringa” para essa condição associada à obesidade. Embora ainda seja necessária a busca por novas opções terapêuticas que reduzam, de fato, a mortalidade geral nesse contexto, esses novos tratamentos impactaram efetivamente a diminuição da hospitalização e dos sintomas desses pacientes. Por isso, inicia-se uma nova era no manejo da insuficiência cardíaca.

## Graphical abstract

**Figura Central:**
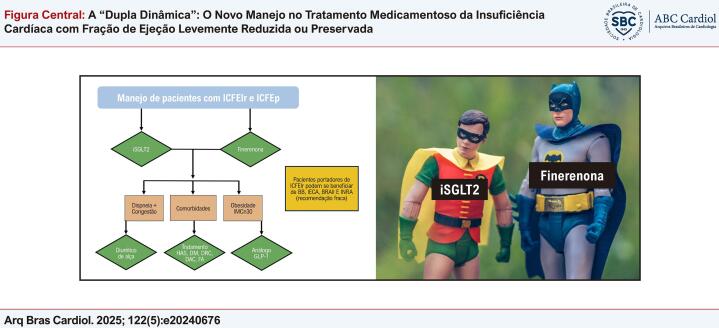
A “Dupla Dinâmica”: O Novo Manejo no Tratamento Medicamentoso da Insuficiência Cardíaca com Fração de Ejeção Levemente Reduzida ou Preservada

## Introdução

A insuficiência cardíaca (IC) é definida, de forma universal, como a presença de sinais e/ou sintomas de IC causados por alterações estruturais ou funcionais cardíacas, associados a pelo menos um dos seguintes fatores: elevação dos peptídeos natriuréticos ou evidência de congestão pulmonar ou sistêmica.^1^ Essa condição apresenta uma expressiva prevalência, atingindo em torno de 23 milhões de pessoas no mundo,^2^ especialmente entre idosos, acometendo mais de 10% dos indivíduos acima dos 70 anos. Ressalta-se a elevada mortalidade da IC, que pode chegar a 67% em até cinco anos após o diagnóstico,^3^ apresentando, inclusive, pior prognóstico em relação a algumas neoplasias malignas.^4^

A classificação da IC é determinada de acordo com a fração de ejeção do ventrículo esquerdo (FEVE) e sua importância se baseia nos diferentes prognósticos e tratamentos de cada grupo. Nesse sentido, a IC com fração de ejeção reduzida (ICFEr) é aquela com FE ≤ 40%, enquanto a IC com fração de ejeção preservada (ICFEp) e IC com fração de ejeção preservada levemente reduzida (ICFElr) apresentam, respectivamente, FE ≥ 50% e FE entre 41-49%. Quanto à prevalência, a ICFEr é a mais comum (60%), seguida da ICFElr (24%) e ICFEp (16%).^3^ Já em relação ao prognóstico, portadores de FEVE reduzida apresentam maior mortalidade anual (8,8%) se comparados à FEVE preservada (6,3%).

O tratamento medicamentoso reduziu, de forma significativa, a morbimortalidade nos indivíduos com IC. Contudo, por muito tempo, o benefício medicamentoso modificador da doença se restringiu à ICFEr. Assim, a terapia medicamentosa baseada nos quatro pilares — (1) inibidores da enzima conversora de angiotensina (IECA)/bloqueadores do receptor da angiotensina II (BRA)/inibidores da neprilisina e receptor da angiotensina II (INRA), (2) betabloqueadores (BB), (3) antagonistas de mineralocorticoides (ARM) e (4) inibidores do cotransportador de sódio e glicose II (iSGLT2) — mudou expressivamente o curso da ICFEr, reduzindo em 64% a mortalidade cardiovascular (CV) e a hospitalização por IC nesses pacientes.^5^ Nesse sentido, o chamado “Quarteto Fantástico”, termo inicialmente mencionado em 2021^6^ e consagrado desde então, fez jus ao nome em pacientes com FEVE reduzida, o que não se replicou de forma satisfatória nos portadores de ICFElr e ICFEp, nos quais, por muitos anos, o tratamento medicamentoso careceu de benefícios consistentes na redução da mortalidade e hospitalização.^7^

**Figura 1:**
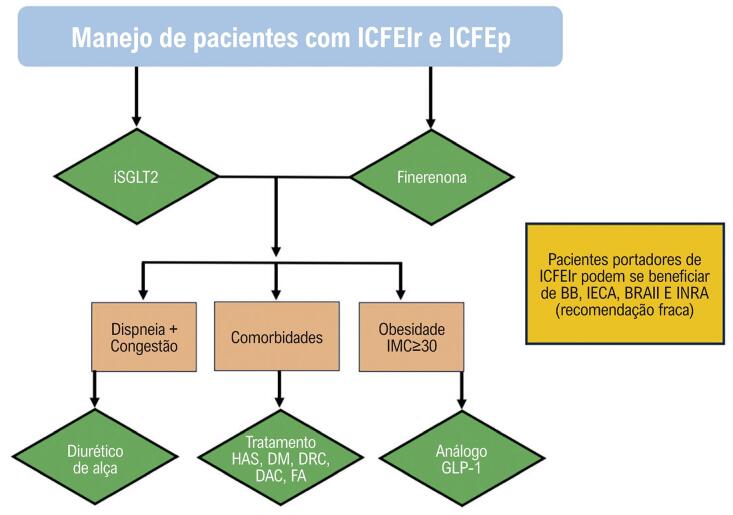
– Sugestão de manejo da insuficiência cardíaca com fração de ejeção levemente reduzida ou preservada. BB: betabloqueador; BRAII: bloqueador do receptor de angiotensina II; DAC: doença arterial coronariana; DM: diabetes mellitus; DRC: doença renal crônica; FA: fibrilação atrial; GLP-1: receptor de peptídeo 1 semelhante ao glucagon; HAS: hipertensão arterial sistêmica; ICFElr: insuficiência cardíaca com fração de ejeção levemente reduzida; ICFEp: insuficiência cardíaca com fração de ejeção preservada; IECA: inibidor da enzima conversora de angiotensina; IMC: índice de massa corporal; INRA: inibidor da neprilisina e receptor da angiotensina; iSGLT2: inibidores do cotransportador de sódio e glicose II.

No entanto, diante de novas evidências, a partir de 2021, sobretudo em relação aos iSGLT2 e aos novos antagonistas de mineralocorticoides (Finerenona), houve, por fim, a concretização de um tratamento com efetiva redução do desfecho composto de morte cardiovascular e hospitalização por IC (às custas deste último) nos pacientes com FEVE superior a 40% (Figura Central), levando ao surgimento de um novo termo: a “Dupla Dinâmica” da ICFElr e ICFEp (Figura 1).

Assim sendo, o presente artigo tem como o objetivo revisar, descrever e sugerir as recentes possibilidades terapêuticas que mostraram benefícios concretos na IC com FEVE>40%, demonstrando e analisando as evidências disponíveis na literatura, bem como explorando os possíveis mecanismos fisiopatológicos envolvidos.

### Quarteto Fantástico: O tratamento da ICFEr e ICFElr/ICFEp

A ICFEr apresenta uma complexa fisiopatologia, deflagrada após injúria miocárdica (primária e/ou secundária), que ativa, por sua vez, o sistema neuro-hormonal, composto pelo sistema renina-angiotensina-aldosterona (SRAA) e pelo sistema nervoso simpático (SNS). Se, por um lado, esse mecanismo adaptativo visa manter o débito cardíaco às custas de inotropismo, cronotropismo e otimização da pré-carga em paralelo à manutenção da perfusão tecidual através da vasoconstrição, por outro, leva à hipervolemia, inflamação sistêmica, remodelamento adverso e progressão da IC.^8^

Nesse contexto, o “Quarteto Fantástico” atua justamente nas vias fisiopatológicas da ICFEr, atenuando o ciclo progressivo da IC, reduzindo morbimortalidade e, portanto, apresenta recomendação classe I pelas principais diretrizes, nacional e internacionais, para o manejo de FEVE reduzida.^3,9,10^

No entanto, o tratamento medicamentoso na FEVE levemente reduzida ou preservada não manteve o mesmo “heroísmo” evidenciado na ICFEr, como destacado por muitos estudos falhos (Tabela 1).

**Tabela 1:** – Estudos randomizados na avaliação de tratamento específico para insuficiência cardíaca com fração de ejeção levemente reduzida ou preservada

Estudo (ano)	Droga (classe)	Amostra Seguimento (média ou mediana)	Inclusão	Morte CV e HR ou RR hospitalização IC (IC de 95%)	Morte geral HR (IC de 95%)	Morte Cardiovascular HR (IC de 95%)	Hospitalização IC HR ou RR (IC de 95%)
Metanálise Cleland et al. (2018)^15^	BB	n=14.262 (ritmo sinusal)/15,3 meses	Ritmo sinusal; IC; qualquer FE (n=575, FE-41-49%) (n=244, FE≥50%)	FE=40-49%: 0,83 (0,6-1,13) FE≥50%: 0,66 (0,38-1,15)	FE=40-49%: 0,59 (0,34-1,03) FE≥50%: 1,79 (0,78-4,1)	FE=40-49%: 0,48 (0,24-0,97) FE≥50%: 1,77 (0,61-5,14)	FE=40-49%: 0,95 (0,68-1,32) FE≥50%: 0,66 (0,37-1,18)
PEP-CHF (2006)^17^	Perindopril (ECA)	n=850/26,2 meses	Idade≥70 anos; IC sintomática; uso de diuréticos; disfunção diastólica; ausência de disfunção sistólica	0,92 (0,70-1,21)*	1,09 (0,75-1,58)	0,98 (0,63-1,53)	0,86 (0,61-1,20)
CHARM-Preserved (2003)^18^	Candesartana (BRAII)	n=3.023/36,6 meses	IC sintomática; FE≥40%; internação por causa cardíaca prévia	0,86 (0,74-1,00)	NR	0,95 (0,76-1,18)	0,84 (0,70-1,00)
PARAGON-HF (2019)^20^	Sacubitril/Valsartana (INRA)†	n=4.822/35 meses	Idade≥50 anos; IC sintomática; uso de diuréticos; FE≥45%; alteração estrutural cardíaca; elevação BNP/NT pro-BNP	0,87 (0,75-1,01)	0,97 (0,84-1,13)	0,95 (0,79-1,16)	0,85 (0,72-1,00)
TOPCAT (2014)^22^	Espironolactona (ARM)	n=3.445/39 meses	Idade≥50 anos; IC sintomática; FE≥45%; elevação BNP/NT pro-BNP ou hospitalizações ≤12 meses	0,89 (0,77-1,04)	0,91 (0,77-1,08)	0,90 (0,73-1,12)	0,83 (0,69-0,99)
FINEARTS-HF (2024)^37^	Finerenona (ARM não esteroidal)	n=6.001/32 meses	Idade≥40 anos; IC sintomática; uso de diuréticos, FE≥40%; alteração estrutural cardíaca; elevação BNP/NT pro-BNP	0,84 (0,74-0,95)	0,93 (0,83-1,06)	0,93 (0,78-1,11)	0,82 (0,71-0,94)
EMPEROR-Preserved (2021)^29^	Empagliflozina (ISGLT2)	n=5.988/26,2 meses	Idade≥18 anos; IC sintomática; FE≥40%; alteração estrutural cardíaca; elevação BNP/NT pro-BNP	0,79 (0,69-0,90)	1,00 (0,87-1,15)	0,91 (0,76-1,09)	0,71 (0,60-0,83)
DELIVER (2022)^30^	Dapagliflozina (ISGLT2)	n=6.263/27,6 meses	Idade≥40 anos; IC sintomática; FE≥40% ou melhorada; alteração estrutural cardíaca; elevação BNP/NT pro-BNP	0,82 (0,73-0,92)	0,94 (0,83-1,07)	0,88 (0,74-1,05)	0,77 (0,67-0,89)
Metanálise Butler et al. (2024)^44^	Semaglutida (análogo GLP-1)	n=1.145/13 meses	Idade≥18 anos; IMC≥30 kg/m^2^; IC sintomática; FE≥45%; KCCQ≤90; TC6≥100 m; aumento pressões enchimento VE com alteração estrutural cardíaca e elevação BNP/NT pro-BNP ou internação por IC e uso de diurético com BNP/NT pro-BNP elevado	0,31 (0,15-0,62)	NR	NR	0,27 (0,12-0,56)

*Desfecho de morte geral e hospitalização por IC; †Diferentemente dos outros estudos citados (que compararam o tratamento com placebo), o estudo PARAGON-HF comparou sacubitril/valsartana com valsartana; ‡Desfecho incluiu morte CV, Hospitalização por IC e parada cardíaca abortada. ARM: antagonista receptor mineralocorticoide; BB: betabloqueador; BNP: peptídeo natriurético tipo B; BRAII: bloqueador do receptor de angiotensina II; CV: cardiovascular; FE: fração de ejeção; GLP-1: receptor de peptídeo 1 semelhante ao glucagon; HR: hazard ratio; IC: insuficiência cardíaca; IC: intervalo de confiança; IECA: inibidor da enzima conversora de angiotensina; IMC: índice de massa corporal; INRA: inibidor da neprilisina e receptor da angiotensina; iSGLT2: inibidores do cotransportador de sódio e glicose II; KCCQ: Kansas City Cardiomyopathy Questionnaire; NR: não registrado; NT pro-BNP: N-terminal do peptídeo natriurético tipo B; RR: rate ratio; TC6: teste de caminhada de 6 minutos.

Inicialmente, os betabloqueadores, que reduziram em torno de 31-34% da mortalidade geral na FEVE reduzida,^11-14^ nunca apresentaram a mesma consistência em FE superiores a 40%. Apesar de poucos estudos randomizados com BB nesse contexto, uma metanálise publicada em 2018, que avaliou o uso de BB em 14.262 pacientes portadores de IC em ritmo sinusal (com todos os espectros de FEVE), não evidenciou redução na mortalidade geral nos pacientes com FE de 40-49% (HR=0,59; IC de 95%: 0,34–1,03, p=0,066) e naqueles com FE preservada (HR=1,79; IC de 95%: 0,78–4,1, p=0,17).^15^

A inibição do sistema renina-angiotensina II também não se mostrou tão favorável como nos estudos randomizados em portadores de ICFEr, onde o IECA alcançou redução de mortalidade muito considerável, na ordem de 40%.^16^ Nesse sentido, em avaliação do perindopril nos pacientes com disfunção diastólica (sem disfunção sistólica) e necessidade de diurético, a sobrevida não se modificou nesse perfil de indivíduos (HR=0,919; IC de 95%: 0,700-1,208; p=0,545).^17^ Além disso, em relação ao BRA II, o conhecido estudo CHARM-Preserved também falhou em demonstrar eficácia na redução de morte e hospitalização em FEVE superior a 40%.^18^ Ainda, os INRA, cujo ensaio clínico randomizado prévio em pacientes com FEVE reduzida (PARADIGM-HF) demonstrou redução de mortalidade em 16% sobre o IECA,^19^ também foram decepcionantes no que diz respeito à FE≥45%, onde não se obteve redução de desfecho de morte/hospitalização.^20^

Seguindo ainda o roteiro de desfechos não significativos no cenário da ICFElr e ICFEp, os ARM, diferentemente do que demonstraram na ICFEr (onde reduziram em 30% a mortalidade geral)^21^ também não foram eficazes na redução do desfecho composto de morte cardiovascular/hospitalização/morte súbita abortada, quando avaliados na IC com FE≥45%.^22^

Por isso, diante do insucesso do “Quarteto Fantástico” na conjuntura da ICFElr/ICFEp, as diretrizes recomendaram, para o tratamento da IC com FEVE preservada, por muitos anos, apenas o uso de diuréticos para sintomas e o controle das comorbidades, como a obesidade, hipertensão, diabetes, fibrilação atrial e isquemia miocárdica.^3,10^

**Figura 2:**
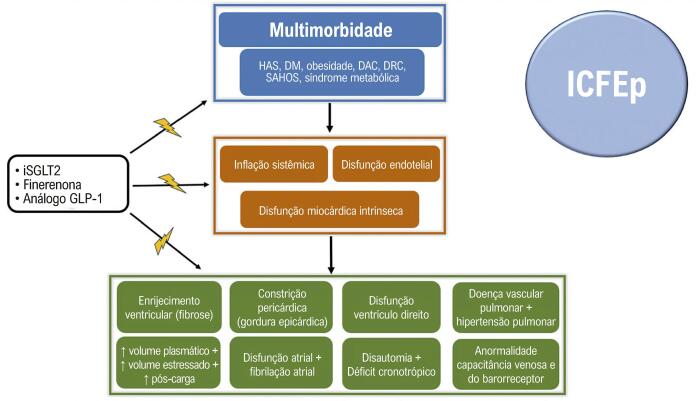
– Fisiopatologia da insuficiência cardíaca com fração de ejeção preservada e os alvos terapêuticos dos tratamentos atuais disponíveis. DAC: doença arterial coronariana; DM: diabetes mellitus; DRC: doença renal crônica; GLP-1: receptor de peptídeo 1 semelhante ao glucagon; HAS: hipertensão arterial sistêmica; ICFEp: insuficiência cardíaca com fração de ejeção preservada; SAHOS: síndrome da apneia e hipopneia obstrutiva do sono; iSGLT2: inibidores do cotransportador de sódio e glicose II.

O fracasso está relacionado a profundas diferenças na fisiopatologia entre a ICFEr e a ICFEp. Isso porque, enquanto a primeira está intimamente relacionada ao baixo débito e à cascata neuro-hormonal, a segunda é resultado de mecanismos fisiopatológicos complexos, heterogêneos e ainda não tão completamente compreendidos. Na verdade, os contrastes vão além da disfunção sistólica e diastólica, uma vez que ambas as alterações estão presentes, independente da FEVE.^23^ Assim, a ICFEp é resultado de intensas alterações fisiopatológicas relacionadas, sobretudo, à inflamação sistêmica, disfunção endotelial, alterações energéticas do miocárdio e volemia, reflexos esses da multimorbidade (obesidade, hipertensão, diabetes e síndrome metabólica) (Figura 2).^24^

Portanto, na ICFEp, são múltiplas as consequências encontradas pelos diversos mecanismos envolvidos na doença, dentre eles: o enrijecimento ventricular (fibrose); constrição pericárdica (gordura epicárdica); aumento do volume plasmático, volume estressado e da pós-carga; disfunção do ventrículo direito; doença vascular pulmonar e hipertensão pulmonar; disfunção atrial e fibrilação atrial; disautonomia e déficit cronotrópico; anormalidade da capacitância venosa e baixa sensibilidade dos barorreceptores. Fica compreensível, dessa maneira, o quanto a complexa e singular fisiopatologia da ICFEp difere da ICFEr e, por isso, pode-se justificar diferenças em seus tratamentos.^23,24^

Apesar de que, até pouco tempo, não havia nenhum medicamento que reduzisse efetivamente o desfecho na IC com FEVE>40%, a partir do ano de 2021 iniciou-se uma nova era no manejo da ICFElr/ICFEp com os iSGLT2 e, mais recentemente, em 2024, com os novos antagonistas de mineralocorticoides, a finerenona.

### A dupla dinâmica: iSGLT2 e Finerenona

#### iSGLT2:

Os iSGLT2, representados mais comumente pela dapagliflozina e empagliflozina, atuam inibindo a reabsorção de glicose no túbulo contorcido proximal do néfron, causando glicosúria e, consequentemente, melhor controle glicêmico, tendo sido desenvolvidos, inicialmente, para o tratamento de DM2. Em estudos de segurança, notou-se redução de internação por IC em torno de 30%,^25^ o que propiciou, em um primeiro momento, a publicação de estudos relacionados ao uso dos iSGLT2 no contexto específico de ICFEr, independente da DM2 (EMPEROR-Reduced e DAPA-HF representando, respectivamente, empagliflozina e dapagliflozina), que levou à redução de 26% morte CV e hospitalização, além de mortalidade geral em 13%.^26-28^

Posteriormente, foi avaliada a eficácia dos iSGLT-2 na redução de desfechos de morte e internação por IC no contexto de ICFElr/ICFEp, também independentemente de DM2. Publicou-se, então, o EMPEROR-Preserved (2021), randomizado e placebo-controlado, que avaliou a empagliflozina em 5.988 indivíduos com IC e FEVE>40%, durante 26,2 meses, e demonstrou redução do desfecho primário em 21%, às custas, sobretudo, de hospitalização.^29^ Apesar de não ter apresentado impacto na mortalidade, foi o primeiro estudo que foi positivo na FEVE preservada. Um ano depois, em 2022, foi publicado estudo DELIVER, que randomizou 6.263 pacientes para avaliação da dapagliflozina no mesmo contexto, por 2,3 anos, e, como resultado, reforçaram-se os mesmos achados da empagliflozina, também reduzindo em 18% o composto de morte CV/hospitalização.^30^ Em metanálise de ambos os estudos citados, houve redução do desfecho composto em 20% (HR=0,80, IC de 95%: 0,73–0,87) e de 26% na internação por IC (HR=0,74, IC de 95%: 0,67–0,83).^31^

Portanto, de maneira surpreendente, após muitos anos sem que houvesse um tratamento específico com impacto na ICFEp, os iSGLT2 foram incorporados na diretrizes internacionais com recomendação forte para o tratamento da ICFElr e ICFEp.^9,32^

Após inúmeros estudos negativos, questiona-se qual seria o racional do impacto dos iSGLT2 na IC com FEVE>40%. Atribui-se, de início, aos mecanismos convencionais que podem, em parte, justificar a melhoria cardiovascular, como aumento da diurese, otimização da hemoglobina glicada, aumento de hematócrito, discreta perda de peso e da pressão arterial. Entretanto, outros tratamentos (antidiabéticos, diuréticos e anti-hipertensivos) podem levar a tais efeitos, sem que levassem aos desfechos positivos apresentados, inferindo, assim, que demais mecanismos, não convencionais, pudessem estar associados ao impacto nos desfechos apresentados. Dentre eles, estão a redução da gordura epicárdica, a autofagia de organelas danificadas, a redução de inflamação e do estresse oxidativo, o aumento do cálcio intracelular (e consequente aumento da contração cardíaca), a otimização energética miocárdica, melhoria da função endotelial e da eficiência cardíaca.^33^ Desse modo, conhecendo-se tais benefícios que atuam diretamente em vários aspectos fisiopatológicos da ICFEp, pode-se compreender melhor o impacto positivo dos iSGLT2 nesses pacientes.

## Antagonistas receptor mineralocorticoides (Finerenona):

Também parte do “Quarteto Fantástico”, os ARM apresentaram previamente resultados limítrofes quanto ao benefício na ICFElr/ICFEp, apresentando, na atualidade, algum grau de recomendação nesse contexto (IIb), pela diretriz Americana de IC publicada em 2022.^9^ Isso porque o estudo TOPCAT (2014), que apesar de não ter apresentado desfecho primário positivo na avaliação do uso da espironolactona em pacientes portadores de IC com FEVE≥45% (amostra total de 3.445), conforme citado previamente, possui algumas observações pertinentes.

Primeiramente, quanto à análise do desfecho secundário de hospitalização, houve redução de 17% (HR=0,83, IC de 95%: 0,69–0,99; p=0,04) o que, no cenário de elevada morbidade e perda de qualidade de vida na IC, já se aponta como um resultado favorável.^22^

Ainda, em análise post hoc, foi verificado que no subgrupo dos pacientes incluídos no estudo provenientes do continente Americano (Estados Unidos, Canadá, Brasil e Argentina, correspondente a 51% amostral), houve redução do desfecho primário (morte CV, hospitalização por IC e morte súbita abortada) em 18% (HR=0,82; IC de 95%: 0,69–0,98, p=0,026), da hospitalização em 18% (HR=0,82; IC de 95%: 0,67–0,99, p=0,042) e, também, na mortalidade CV em 26% (HR=0,74; IC de 95%: 0,57–0,97, p=0,027). Entretanto, ele não foi observado em parte da amostra oriunda da Rússia/Geórgia (49% amostral), onde não existiu qualquer benefício nos desfechos primário ou secundários. Dentre as hipóteses que poderiam justificar tais resultados, destacam-se: diferença significativa das características basais das duas populações analisadas (idade, prevalência de comorbidades, FEVE, dentre outros); maior número de eventos primários ocorridos na população da América (29,5% contra 8,9% na Rússia/Geórgia); número superior de pacientes incluídos no estudo pelo critério relacionado à elevação de BNP na população americana (45%), quando comparado a outra população (11%), que foi, em sua maioria, incluída no estudo devido ao critério de hospitalização prévia (possibilidade de inclusão não acurada de portadores de ICFEp).^34^ Por fim, em outra análise publicada em 2017, a partir 366 indivíduos participantes TOPCAT, notou-se que houve uma proporção superior de pacientes com dosagem indetectável de canrenona (metabólito ativo da espironolactona) nos pacientes da Rússia, quando comparado aos Estados Unidos (30% e 3%, respectivamente, p<0,001), evidenciando que o uso real do ARM variou de acordo com a origem amostral, podendo ter alterado o desfecho final.^35^

Diante de tais achados limítrofes quanto à eficácia da espironolactona em pacientes com ICFElr/ICFEp, uma nova classe de ARM não esteroidal, a Finerenona, foi avaliada nessa conjuntura (estudo FINEARTS-HF). Sabidamente, esse medicamento apresenta algumas vantagens sobre os demais ARMs, visto ter maior potência e seletividade sobre o receptor mineralocorticoide, ausência penetração no sistema nervoso central ou efeitos colaterais sexuais, menor meia-vida e efeito sobre a pressão arterial. Além disso, demonstrou benefício na redução de desfechos cardiovasculares e renais, com segurança, em pacientes com DM2.^36^

O FINEARTS-HF (2024), estudo que avaliou a Finerenona (20-40 mg) em uma amostra de 6.001 pacientes portadores de IC com FEVE≥40% em um seguimento de 32 meses, apresentou desfecho positivo na redução de morte CV e piora de IC (hospitalização/passagem urgente em pronto-socorro) em 16% (RR=0,84; IC de 95%: 0,74–0,95, p=0,007), sobretudo às custas de hospitalização (diminuição em 18%). Apesar do estudo não ter apresentado impacto na mortalidade, seu resultado é bastante favorável, haja vista que seu desfecho primário foi positivo, inclusive, independentemente do uso prévio de iSGLT2, de forma segura e em uma amostra de pacientes bastante sintomáticos (87% em uso de diuréticos de alça).^37^

A Finerenona, portanto, é o segundo tratamento que demonstrou impacto significativo na ICFElr/ICFEp, reforçando o manejo dessa condição clínica de alta morbimortalidade. De fato, em se tratando dos efeitos dos ARM (atuando na redução da congestão, retenção de sódio, disfunção endotelial, inflamação, fibrose e hipertrofia^23^), parecem ser justificáveis as consequências positivas desse antagonista de mineralocorticoide na ICFEp, principalmente diante de sua atuação em várias vias fisiopatológicos específicas desse padrão singular da IC. Somado a isso, avaliações prévias já haviam mostrado o papel dos ARMs na reversão de alterações estruturais (remodelamento reverso) e na função diastólica cardíaca.^38^

## Semaglutida como tratamento emergente “coringa” no fenótipo da ICFElr/ICFEp e obesidade:

A semaglutida, da classe de medicamentos agonistas do receptor de peptídeo 1 semelhante ao glucagon (GLP-1), já é muito bem estabelecida e recomendada no tratamento DM2, com redução associada de 26% nos desfechos CV na dose de 1,0 mg/semana (morte CV, infarto do miocárdio e acidente vascular encefálico).^39^ Posteriormente, os análogos de GLP-1 ganharam destaque também no tratamento da obesidade, na dose alvo de 2,4 mg/semanal, com diminuição considerável de peso nesse perfil de pacientes, sobretudo no que diz respeito à semaglutida, que proporcionou perda de 12,7 kg em 17 meses,^40^ sendo mantido o benefício em 26 meses (perda de 12,9 kg), em pacientes sem diabetes.^41^ Inclusive, o uso desse análogo de GLP-1 em obesos (mesmo sem DM2) demonstrou, ainda, associado ao emagrecimento, atenuação em 20% dos desfechos CV.^42^

Sabe-se que o sobrepeso/obesidade, conforme previamente descrito, apresenta íntima relação com a IC com FEVE preservada, principalmente devido à inflamação sistêmica consequente dessa comorbidade, estando presente em até 80% dos portadores de ICFEp.^1^ Nesse sentido, o tratamento dessa IC com a semaglutida, em portadores de índice de massa corpórea (IMC) elevada, parece ser racionalmente benéfico, o que é reforçado ao compreender que esse medicamento está associado à proteção cardíaca (redução da inflamação e injúria isquêmica miocárdicas, com aumento da função ventricular, frequência cardíaca e melhora da produção energética através da glicose) e vascular (redução da inflamação endotelial, da proliferação do muscular lisa e da agregação plaquetária, paralelamente à otimização da vasodilatação, do fluxo sanguíneo e da proteção endotelial), atuando, portanto, de forma significativa em mecanismos fisiopatológicos da ICFEp.

Avaliou-se, nesse contexto, em estudo randomizado e duplo-cego (STEP-HFpEF, publicado em 2023), a utilização da semaglutida 2,4 mg/semana em pacientes portadores de IC com FEVE≥45% e IMC≥30 (n=529), sem DM, por 52 semanas. Observou-se redução de 10,7% do peso corporal, associado à melhoria de qualidade de vida e ao aumento da distância no teste de caminhada de 6 minutos (TC6). De interesse, houve redução em 39% da proteína C reativa (PCR), inferindo redução da inflamação sistêmica, e queda de 16% dos peptídeos natriuréticos (BNP), sugerindo compensação da IC.^43^ Um ano depois, foi publicado ensaio clínico com desenho semelhante, porém com amostra composta por 616 pacientes diabéticos (STEP-HFpEF DM), que tendem a apresentar maior gravidade de IC, perdem menos peso na vigência dos análogos de GLP-1 e utilizam, com maior frequência, os iSGLT-2 (35% na amostra). Novamente, apesar de 40% a menos em relação ao estudo prévio, ainda assim houve perda de 6,4% de peso, mantendo incremento da qualidade de vida, da capacidade funcional (TC6), associado a queda de BNP e PCR, mesmo naqueles em uso dos iSGLT-2.

Apesar dos desfechos primários avaliados terem sido a perda de peso e qualidade de vida, em metanálise, contendo amostra de ambos os estudos (n=1145), incluindo pacientes com e sem DM, percebe-se uma redução de morte CV e eventos por IC (hospitalização e passagem urgente por IC) em 69% a favor do uso da semaglutida (HR=0,31, IC de 95%: 0,15–0,62, p=0,0008), com expressiva redução isolada de eventos por IC (HR=0,27, IC de 95%: 0,12–0,56, p=0,0004). O benefício foi mais expressivo naqueles pacientes com BNP mais elevado, em uso de diuréticos e em portadores de fibrilação atrial, ou seja, naqueles com IC clinicamente mais relevante.^44^ Portanto, a hipótese é de que o efeito da semaglutida seja independente apenas da perda de peso, atuando na fisiopatologia da ICFEp, reduzindo a inflamação sistêmica (queda de PCR) e compensando a IC objetivamente, o que se reflete na queda dos peptídeos natriuréticos (sendo que a perda de peso isolada tende a elevar o BNP, como se vê em estudos com pacientes em pós-operatório de cirurgia bariátrica).^45^ Assim, a ICFEp, na vigência da obesidade, parece ter a semaglutida como tratamento “coringa” e eficaz.

## Conclusão

Por tudo isso, após muitos anos sem tratamento específico de impacto na ICFElr/ICFEp, dois medicamentos recentemente estão mudando o rumo do manejo dessa condição clínica, os iSGLT2 e a Finerenona, a “Dupla dinâmica” que, de forma análoga ao “Quarteto Fantástico” na ICFEr, terão recomendação conjunta instituída pelas diretrizes na terapia de IC com FEVE>40%. A semaglutida, por sua vez, parece apresentar benefícios nesse contexto associado à obesidade, podendo ser, assim, indicada.

Apesar de animador, ainda são necessárias novas publicações almejando tratamentos que apresentem impacto definitivo na mortalidade. Entretanto, diante de uma condição clínica que, por muitos anos, não apresentava redução de desfechos perante o principal arsenal terapêutico existente, a “Dupla Dinâmica” representa uma reviravolta no combate à morbimortalidade desse espectro particular da IC.
