# Supplementary material for: A “Dupla Dinâmica”: O Novo Manejo no Tratamento Medicamentoso da Insuficiência Cardíaca com Fração de Ejeção Levemente Reduzida ou Preservada
Source: Arq Bras Cardiol. 2025 May 8;122(5):e20240676. [Article in Portuguese] doi: 10.36660/abc.20240676 (PMC12129481; doi:10.36660/abc.20240676)
Supplement: Translation

# Translation: “The Dynamic Duo”: The New Management of Drug Treatment for Heart Failure with Mildly Reduced or Preserved Ejection Fraction

**DOI:** 10.36660/abc.20240676i

**Authors:** Plínio José Whitaker Wolf, Edileide Barros Correia, João Manoel Rossi, Marco Aurelio Finger, Carolina Casadei Santos, Marcos de Oliveira Vasconcellos, Larissa Ventura Ribeiro Bruscky, Ana Cristina de Souza Murta, Yoná Afonso Francisco, Fabiano Castro Albrecht, Juliana Jangelavicin Barbosa, Eduardo Mikio Sassaki, Bruno Noshang Blaas, Bianca Fernandes Távora Arruda, Fernanda de Brito Fortuna, Victor Bemfica de Mello Mattos

**Affiliations:** 1 Brazil Instituto Dante Pazzanese de Cardiologia, São Paulo, SP – Brazil; 2 Brazil Centro de Referência e Treinamento DST/Aids-SP, São Paulo, SP – Brazil; 3 Brazil Escola Paulista de Medicina da Universidade Federal de São Paulo, São Paulo, SP – Brazil

**Keywords:** Heart Failure, Sodium-Glucose Transporter 2 Inhibitors, Mineralocorticoid Receptor Antagonists

## Abstract

The “Fantastic Four,” a term coined in 2021 to refer to the four key drug pillars in the treatment of heart failure with reduced ejection fraction (beta-blockers, renin-angiotensin system and neprilysin inhibitors, mineralocorticoid receptor antagonists, and sodium-glucose cotransporter-2 inhibitors, or SGLT2 inhibitors), has demonstrated excellent performance in reducing morbidity and mortality in this setting. However, in heart failure with mildly reduced or preserved ejection fraction, the same benefits were not observed with this combined treatment where, for many years, management in this context was limited to diuretics and comorbidity control. Recently, however, new therapeutic options have emerged, demonstrating effectiveness in reducing cardiovascular outcomes in this specific group: the “Dynamic Duo”—comprising SGLT2 inhibitors and Finerenone—has shown promising results, alongside the introduction of semaglutide as a potential “wild card” treatment for patients with obesity. Despite the ongoing need for therapies that significantly reduce overall mortality, these new treatments have effectively lowered hospitalization rates and improved symptoms in such patients. As a result, a new era in heart failure management is beginning.

## Introduction

Heart failure (HF) is universally defined as the presence of signs and/or symptoms caused by structural or functional cardiac abnormalities, along with at least one of the following: elevated natriuretic peptides or evidence of pulmonary or systemic congestion.^1^ This condition has a significant global prevalence, affecting approximately 23 million people around the world,^2^ particularly older people, with more than 10% of individuals over 70 years of age being impacted. Notably, HF has a high mortality rate, reaching 67% within five years of diagnosis,^3^ and has a worse prognosis of some malignant neoplasms.^4^

HF is classified based on left ventricular ejection fraction (LVEF), which is essential for determining prognosis and treatment. In this sense, HF with reduced ejection fraction (HFrEF) is defined by EF ≤ 40%, while HF with preserved ejection fraction (HFpEF) and HF with mildly reduced ejection fraction (HFmrEF) correspond to EF ≥ 50% and EF between 41–49%, respectively. Regarding prevalence, HFrEF is the most frequent (60%), followed by HFmrEF (24%) and HFpEF (16%).^3^ In relation to prognosis, patients with reduced LVEF have a higher annual mortality rate (8.8%) compared to those with preserved LVEF (6.3%).

Drug treatment has significantly reduced morbidity and mortality in individuals with HF. However, for a long time, the disease-modifying benefits of drugs were limited to HFrEF. Thus, drug therapy based on four pillars—(1) angiotensin-converting enzyme inhibitors (ACEI)/angiotensin II receptor blockers (ARBs)/angiotensin II receptor neprilysin inhibitors (ARNIs), (2) beta-blockers (BBs), (3) mineralocorticoid antagonists (MRAs) and (4) sodium-glucose cotransporter II inhibitors (SGLT2 inhibitor)—has significantly changed the course of HFrEF, reducing cardiovascular (CV) mortality and hospitalization for HF in these patients by 64%.^5^ In this sense, the so-called “Fantastic Four,” a term initially coined in 2021^6^ and consecrated since then, lived up to its name in those patients with reduced LVEF, which was not satisfactorily replicated in those with HFmrEF and HFpEF, where drug treatment lacked, for many years, consistent benefits in terms of reducing mortality and hospitalization.^7^

Since 2021, new evidence, particularly regarding SGLT2 inhibitors and mineralocorticoid antagonists (Finerenone), has led to a breakthrough in treating patients with LVEF >40% (Central Illustration). These therapies have demonstrated a meaningful reduction in the composite outcome of cardiovascular death and HF-related hospitalizations (primarily by reducing hospitalizations), which has given rise to the “Dynamic Duo” for managing HFmrEF and HFpEF (Figure 1).

**Figure 1:** – Suggested management of heart failure with mildly reduced or preserved ejection fraction. BB: beta-blocker; ARBs: angiotensin II receptor blocker; CAD: coronary artery disease; DM: diabetes mellitus; CKD: chronic kidney disease; AF: atrial fibrillation; GLP-1: glucagon-like peptide-1 receptor; SAH: systemic arterial hypertension; HFmrEF: heart failure with mildly reduced ejection fraction; HFpEF: heart failure with preserved ejection fraction; ACEI: angiotensin-converting enzyme inhibitor; BMI: body mass index; ARNI: angiotensin receptor neprilysin inhibitor; SGLT2 inhibitors: sodium-glucose cotransporter II inhibitors.

Therefore, this article aims to review, describe and suggest recent therapeutic possibilities that have shown solid benefits in HF with LVEF>40% by demonstrating and analyzing the evidence available in the literature, as well as exploring the possible pathophysiological mechanisms involved.

### Fantastic Four: The treatment of HFrEF and HFmrEF/HFpEF

HFrEF presents a complex pathophysiology triggered by myocardial injury (primary and/or secondary) that activates, in turn, the neurohormonal system, composed of the renin-angiotensin-aldosterone system (RAAS) and the sympathetic nervous system (SNS). If, on the one hand, such an adaptive mechanism aims to maintain cardiac output at the expense of inotropism, chronotropism and preload optimization in parallel with the maintenance of tissue perfusion through vasoconstriction, on the other hand, it leads to hypervolemia, systemic inflammation, adverse remodeling, and progression of HF.^8^

In this setting, the “Fantastic Four” targets the pathophysiological mechanisms of HFrEF, interrupting the progressive HF cycle and reducing morbidity and mortality, thus holding a Class I recommendation in major national and international guidelines for managing reduced LVEF.^3,9,10^

However, drug treatment in mildly reduced or preserved LVEF did not maintain the same “heroism” demonstrated in HFrEF, as highlighted by many unsuccessful studies (Table 1).

**Table 1:** – Randomized studies evaluating specific treatment for Heart Failure with mildly reduced or preserved ejection fraction

Study (year)	Drug (class)	Sample Follow-up (mean or median)	Inclusion	CV death and HR or RR hospitalization HF (95% CI)	Overall death HR (95% CI)	Cardiovascular death HR (95% CI)	HF Hospitalization HR or RR (95% CI)
Cleland et al. meta-analysis (2018)^15^	BB	n=14,262 (sinus rhythm)/ 15.3 months	Sinus rhythm; HF; any EF (n=575, EF-41-49%) (n=244, EF≥50%)	EF=40-49%: 0.83 (0.6-1.13) EF ≥50%: 0.66 (0.38-1.15)	EF= 40-49%: 0.59 (0.34-1.03) EF ≥50%: 1.79 (0.78-4.1)	EF= 40-49%: 0.48 (0.24-0.97) EF≥50%: 1.77 (0.61-5.14)	EF= 40-49%: 0.95 (0.68-1.32) EF ≥50%: 0.66 (0.37-1.18)
PEP-CHF (2006)^17^	Perindopril (ACEI)	n=850/26.2 months	Age≥70 years; symptomatic HF; use of diuretics; diastolic dysfunction; absence of systolic dysfunction	0.92 (0.70-1.21)*	1.09 (0.75-1.58)	0.98 (0.63-1.53)	0.86 (0.61-1.20)
CHARM-Preserved (2003)^18^	Candesartan (BRAI)	n=3,023/ 36.6 months	Symptomatic HF; EF≥40%; hospitalization for previous cardiac cause	0.86 (0.74-1.00)	NR	0.95 (0.76-1.18)	0.84 (0.70-1.00)
PARAGON-HF (2019)^20^	Sacubitril/Valsartan (ARNI)†	n=4,822/35 months	Age ≥50 years; Symptomatic HF; use of diuretics; EF≥45%; cardiac structural alteration; elevated BNP/NT pro-BNP	0.87 (0.75-1.01)	0.97 (0.84-1.13)	0.95 (0.79-1.16)	0.85 (0.72-1.00)
TOPCAT (2014)^22^	Spironolactone (MRA)	n=3,445/39 months	Age≥50 years; symptomatic HF; EF≥45%; elevated BNP/NT pro-BNP or hospitalizations ≤12 months	0.89 (0.77-1.04)	0.91 (0.77-1.08)	0.90 (0.73-1.12)	0.83 (0.69-0.99)
FINEARTS-HF (2024)^37^	Finerenone (non-steroidal MRA)	n=6,001/32 months	Age≥40 years; symptomatic HF; use of diuretics; EF≥40%; cardiac structural alteration, elevated BNP/NT pro-BNP	0.84 (0.74-0.95)	0.93 (0.83-1.06)	0.93 (0.78-1.11)	0.82 (0.71-0.94)
EMPEROR-Preserved (2021)^29^	Empagliflozin (SGLT2 inhibitors)	n=5,988/ 26.2 months	Age ≥18 years; IC: EF≥40%; cardiac structural alteration; elevated BNP/NT pro-BNP	0.79 (0.69-0.90)	1.00 (0.87-1.15)	0.91 (0.76-1.09)	0.71 (0.60-0.83)
DELIVER (2022)^30^	Dapagliflozin (SGLT2 inhibitors)	n=6,263/ 27.6 months	Age≥40 years; symptomatic HF; EF≥40% οr improved; cardiac structural alteration; elevated BNP/NT pro-BNP	0.82 (0.73-0.92)	0.94 (0.83-1.07)	0.88 (0.74-1.05)	0.77 (0.67-0.89)
Butler et al. meta-analysis (2024)^44^	Semaglutide (GLP-1 agonist)	n=1,145/13 months	Age ≥18 years; BMI≥30 kg/m^2^; symptomatic HF; EF≥45%; KCCQ≤90; 6MWT≥100 m; increased LV filling pressures with cardiac structural alteration and elevated BNP/NT pro-BNP or hospitalization for HF and use of diuretic with elevated BNP/NT pro-BNP	0.31 (0.15-0.62)	NR	NR	0.27 (0.12-0.56)

^*^Outcome of overall death and hospitalization for HF; ^†^Unlike the other referenced studies (which compared treatment with placebo), the PARAGON-HF study compared sacubitril/valsartan with valsartan; ^‡^Outcome included CV death, hospitalization due to HF, and aborted cardiac arrest. MRA: mineralocorticoid receptor antagonist; BB: beta-blocker; BNP: B-type natriuretic peptide; ARBs: angiotensin II receptor blocker; CV: cardiovascular; EF: ejection fraction; GLP-1: glucagon-like peptide 1 receptor; HR: hazard ratio; HR: hazard ratio; CI: confidence interval; ACEI: angiotensin-converting enzyme inhibitor; BMI: body mass index; ARNI: angiotensin receptor neprilysin inhibitor; SGLT2 inhibitors: sodium-glucose cotransporter II inhibitors; KCCQ: Kansas City Cardiomyopathy Questionnaire; NR: not recorded; NT pro-BNP: N-terminal B-type natriuretic peptide; RR: rate ratio; 6MWT: 6-minute walk test.

Initially, beta-blockers, which reduced the overall mortality by around 31-34% in reduced LVEF,^11^ never showed the same consistency in EFs above 40%. Despite few randomized studies with BB in this context, a meta-analysis published in 2018, evaluating the use of BB in 14,262 patients with HF in sinus rhythm (with all LVEF spectrums), did not show a reduction in overall mortality in patients with EF of 40-49% (HR=0.59; 95% CI: 0.34–1.03, p=0.066) and in those with preserved EF (HR=1.79; 95% CI: 0.78–4.1, p=0.17).^15^

Inhibition of the renin-angiotensin II system was also not as favorable as in randomized studies in patients with HFrEF, where ACEI achieved a very considerable reduction in mortality, in the order of 40%.^16^ In this sense, an evaluation of perindopril in patients with diastolic dysfunction (without systolic dysfunction) and need for diuretics revealed that survival did not change in this profile of individuals (HR=0.919; 95% CI: 0.700-1.208; p=0.545).^17^ Additionally, in relation to ARB II, the well-known CHARM-Preserved study also failed to demonstrate efficacy in reducing death and hospitalization in LVEF greater than 40%.^18^ Furthermore, the NRAs, whose previous randomized clinical trial in patients with reduced LVEF (PARADIGM-HF) demonstrated a 16% reduction in mortality compared to ACEI,^19^ were also disappointing with regard to EF≥45%, where no reduction in the outcome of death/hospitalization was obtained.^20^

Following the path of non-significant outcomes in the scenario of HFmrEF and HFpEF, the MRAs, unlike what was demonstrated in HFrEF (where they reduced the overall mortality by 30%),^21^ were also not effective in reducing the composite outcome of cardiovascular death/hospitalization/aborted sudden death when evaluated in HF with EF≥45%.^22^

Therefore, given the limited success of the “Fantastic Four” in HFmrEF/HFpEF, guidelines for many years recommended only symptomatic relief with diuretics and comorbidity management—addressing conditions such as obesity, hypertension, diabetes, atrial fibrillation, and myocardial ischemia.^3,10^

This limitation stems from fundamental differences in the pathophysiology of HFrEF and HFpEF. This is because, while the former is closely related to low output and the neurohormonal cascade, the latter is the result of complex, heterogeneous and not yet fully understood pathophysiological mechanisms. In fact, the contrasts go beyond systolic and diastolic dysfunction since both alterations are present, regardless of LVEF.^23^ Thus, HFpEF is the result of intense pathophysiological changes primarily related to systemic inflammation, endothelial dysfunction, myocardial energy changes, and volemia, which are reflections of multimorbidity (obesity, hypertension, diabetes and metabolic syndrome) (Figure 2).^24^

**Figure 2:** – Pathophysiology of heart failure with preserved ejection fraction and therapeutic targets of current treatments available. CAD: coronary artery disease; DM: diabetes mellitus; CKD: chronic kidney disease; GLP-1: glucagon-like peptide 1 receptor; SAH: systemic arterial hypertension; HFpEF: heart failure with preserved ejection fraction; OSAHS: obstructive sleep apnea-hypopnea syndrome; SGLT2 inhibitors: sodium-glucose cotransporter II inhibitors.

Therefore, HFpEF encompasses multiple consequences found by the various mechanisms involved in the disease, including ventricular stiffening (fibrosis); pericardial constriction (epicardial fat); increased plasma volume, stressed volume and afterload; right ventricular dysfunction; pulmonary vascular disease and pulmonary hypertension; atrial dysfunction and atrial fibrillation; dysautonomia and chronotropic deficit; abnormality of venous capacitance and low baroreceptor sensitivity. It is, therefore, understandable how much the complex and unique pathophysiology of HFpEF differs from HFrEF and, therefore, can justify differences in their treatments.^23,24^

Until recently, no medication had effectively improved outcomes in patients with LVEF >40%. However, the landscape shifted in 2021 with the introduction of SGLT2 inhibitors and, more recently, in 2024, with the addition of the new mineralocorticoid antagonist Finerenone.

### The dynamic duo: SGLT2 inhibitors and Finerenone

#### SGLT2 inhibitors:

SGLT2 inhibitors, most commonly represented by dapagliflozin and empagliflozin, act by inhibiting glucose reabsorption in the proximal convoluted tubule of the nephron, causing glycosuria and consequently better glycemic control, having been initially developed for the treatment of DM2. In safety studies, a 30% reduction in HF-related hospitalizations was observed,^25^ prompting the publication of studies on SGLT2 inhibitors use specifically in HFrEF, regardless of DM2 (EMPEROR-Reduced and DAPA-HF representing, respectively, empagliflozin and dapagliflozin), which led to a 26% reduction in cardiovascular death and HF hospitalization, in addition to an overall mortality reduction of 13%.^26^

Subsequent research assessed the efficacy of SGLT2 inhibitors in reducing the outcomes of death and hospitalization due to HF in the context of HFmrEF/HFpEF, also regardless of DM2 EMPEROR-Preserved (2021), a randomized, placebo-controlled study, was then published, evaluating empagliflozin in 5,988 individuals with HF and LVEF >40% for 26.2 months, and demonstrated a 21% reduction in the primary outcome, mainly at the expense of hospitalization.^29^ Although it did not show an impact on mortality, it was the first study that was positive in preserved LVEF. One year later, in 2022, the DELIVER study was published, which randomized 6,263 patients to evaluate dapagliflozin in the same context for 2.3 years, and, as a result, the same findings of empagliflozin were reinforced, also reducing the composite of CV death/hospitalization by 18%.^30^ In a meta-analysis of both studies, there was a 20% reduction in the composite outcome (HR=0.80, 95% CI: 0.73–0.87) and 26% in hospitalization for HF (HR=0.74, 95% CI: 0.67–0.83).^31^

Therefore, surprisingly, after many years without a specific treatment with an impact on HFpEF, SGLT2 inhibitors were incorporated into international guidelines with a strong recommendation for the treatment of HFmrEF and HFpEF.^9,32^

After numerous negative studies, the question arises as to what would be the rationale for the impact of SGLT2 inhibitors in HF with LVEF >40%. Initially, it is attributed to conventional mechanisms that may, in part, justify cardiovascular improvement, such as increased diuresis, optimization of glycated hemoglobin, increased hematocrit, and slight weight loss and blood pressure. However, other treatments (antidiabetic, diuretic, and antihypertensive) may lead to such effects without positive outcomes, thus inferring that other unconventional mechanisms may be associated with the impact on the outcomes presented. Such mechanisms include the reduction of epicardial fat, autophagy of damaged organelles, reduction of inflammation and oxidative stress, increase in intracellular calcium (and consequent increase in cardiac contraction), optimization of myocardial energy, and improvement of endothelial function and cardiac efficiency.^33^ Thus, knowing these benefits, which act directly on several pathophysiological aspects of HFpEF, it is possible to better understand the positive impact of SGLT2 inhibitors on these patients.

## Mineralocorticoid receptor antagonists (Finerenone):

Also, as part of the “Fantastic Four,” MRAs have previously shown borderline results regarding the benefit in HFmrEF/HFpEF, currently presenting some degree of recommendation in this context (IIb) by the American HF guideline published in 2022.^9^ This is because the TOPCAT study (2014), despite not having presented a positive primary outcome in the evaluation of the use of spironolactone in patients with HF with LVEF≥45% (total sample of 3,445), as previously mentioned, has some pertinent observations.

Firstly, regarding the analysis of the secondary outcome of hospitalization, there was a reduction of 17% (HR=0.83, 95% CI: 0.69–0.99; p=0.04), which, in the scenario of high morbidity and loss of quality of life in HF, is already seen as a favorable result.^22^

Furthermore, a post hoc analysis revealed in the subgroup of patients included in the study from the American continent (United States, Canada, Brazil and Argentina, corresponding to 51% of the sample), there was a reduction in the primary outcome (CV death, hospitalization for HF and aborted sudden death) by 18% (HR=0.82; 95% CI: 0.69–0.98, p=0.026), in hospitalization by 18% (HR=0.82; 95% CI: 0.67–0.99, p=0.042) and also in CV mortality by 26% (HR=0.74; 95% CI: 0.57–0.97, p= 0.027). However, the same was not observed in part of the sample from Russia/Georgia (49% of the sample), where there was no benefit in the primary or secondary outcomes. The justifications for such results include the following: significant difference in the characteristics baseline data of the two populations analyzed (age, prevalence of comorbidities, LVEF, among others); a higher number of primary events occurred in the American population (29.5% versus 8.9% in Russia/Georgia); a higher number of patients included in the study by the criterion related to elevated BNP in the American population (45%) when compared to another population (11%), which was mostly included in the study due to the criterion of previous hospitalization (possibility of inaccurate inclusion of patients with HFpEF).^34^ Finally, in another analysis published in 2017, based on 366 individuals participating in TOPCAT, it was noted that there was a higher proportion of patients with undetectable dosages of canrenone (active metabolite of spironolactone) in patients from Russia when compared to the United States (30% and 3%, respectively, p<0.001), showing that the actual use of MRAs varied according to the sample origin, which may have altered the final outcome.^35^

Given such borderline findings regarding the efficacy of spironolactone in patients with HFmrEF/HFpEF, a new class of non-steroidal MRA, Finerenone, was evaluated in this context (FINEARTS-HF study). This drug is known for having some advantages over other MRAs, such as greater potency and selectivity on the mineralocorticoid receptor, lack of penetration into the central nervous system or sexual side effects, shorter half-life and effect on blood pressure. Additionally, it has demonstrated benefits in reducing cardiovascular and renal outcomes safely in patients with DM2.^36^

FINEARTS-HF (2024), a study evaluating Finerenone (20-40 mg) in a sample of 6,001 patients with HF and LVEF≥40% over a 32-month follow-up, showed a positive outcome in reducing CV death and worsening of HF (hospitalization/urgent emergency room visit) by 16% (RR=0.84; 95% CI: 0.74–0.95, p=0.007), mainly at the expense of hospitalization (decrease by 18%). Despite not showing an impact on mortality, the study results are quite favorable, given that its primary outcome was positive, including regardless of prior use of SGLT2 inhibitors, safely and in a sample of very symptomatic patients (87% using loop diuretics).^37^

Finerenone is, therefore, the second treatment that has demonstrated a significant impact on HFpEF/HFpEF, reinforcing the management of this clinical condition with high morbidity and mortality. In fact, when it comes to the effects of MRAs (acting to reduce congestion, sodium retention, endothelial dysfunction, inflammation, fibrosis, and hypertrophy),^23^ the positive consequences of this mineralocorticoid antagonist in HFpEF seem to be justifiable, especially given its action on several specific pathophysiological pathways of this unique pattern of HF. In addition, previous evaluations have already shown the role of MRAs in reversing structural changes (reverse remodeling) and in cardiac diastolic function.^38^

## Semaglutide as an emerging “wild card” treatment for the HFmrEF/HFpEF phenotype and obesity:

Semaglutide, a member of the glucagon-like peptide-1 (GLP-1) receptor agonist class of drugs, is already very well established and recommended for the treatment of T2DM, with an associated reduction of 26% in cardiovascular outcomes at a dose of 1.0 mg/week (CV death, myocardial infarction, and stroke).^39^ Subsequently, GLP-1 agonists also gained prominence in the treatment of obesity, at a target dose of 2.4 mg/week, with considerable weight loss in this patient profile, especially with regard to semaglutide, which provided a loss of 12.7 kg in 17 months,^40^ with the benefit being maintained at 26 months (loss of 12.9 kg) in patients without diabetes.^41^ Furthermore, the use of this GLP-1 agonist in obese individuals (even without DM2) has also been shown, in association with weight loss, to reduce CV outcomes by 20%.^42^

Overweight/obesity, as previously described, is closely related to HF with preserved LVEF, mainly due to the systemic inflammation resulting from this comorbidity, which is present in up to 80% of HFpEF patients.^1^ In this sense, the treatment of this HF with semaglutide in patients with a high Body Mass Index (BMI) appears to be rationally beneficial, which is reinforced by understanding that this medication is associated with cardiac protection (reduction of myocardial inflammation and ischemic injury, with increased ventricular function, heart rate, and improvement in energy production through glucose) and vascular protection (reduction of endothelial inflammation, smooth muscle proliferation, and platelet aggregation, in parallel with the optimization of vasodilation, blood flow, and endothelial protection), therefore acting significantly on the pathophysiological mechanisms of HFpEF.

In this context, a randomized, double-blind study (STEP-HFpEF, published in 2023) evaluated the use of semaglutide 2.4 mg/week in patients with HF with LVEF≥45% and BMI≥30 (n=529), without DM, for 52 weeks. A 10.7% reduction in body weight was observed, associated with an improvement in quality of life and an increase in the distance covered in the 6-minute walk test (6MWT). Of interest, there was a 39% reduction in C-reactive protein (CRP), inferring a reduction in systemic inflammation, and a 16% drop in natriuretic peptides (BNP), suggesting compensation for HF.^43^ A year later, a clinical trial was published with a similar design but with a sample composed of 616 diabetic patients (STEP-HFpEF DM), who tend to have more severe HF, lose less weight while taking GLP-1 agonists and use SGLT-2 inhibitors more frequently (35% in the sample). Again, despite being 40% less than in the previous study, there was still a 6.4% weight loss, maintaining an increase in quality of life and functional capacity (6MWT), associated with a drop in BNP and CRP, even in those using SGLT-2 inhibitors.

Although the primary outcomes were weight loss and quality of life, in a meta-analysis containing a sample from both studies (n=1145), including patients with and without DM, a 69% reduction in CV death and HF events (hospitalization and urgent HF stay) was observed in favor of the use of semaglutide (HR=0.31, 95% CI: 0.15–0.62, p=0.0008), with a significant isolated reduction in HF events (HR=0.27, 95% CI: 0.12–0.56, p= 0.0004). The benefit was more significant in patients with higher BNP, using diuretics, and in those with atrial fibrillation, that is, in those with clinically more relevant HF.^44^ Therefore, the hypothesis is that the effect of semaglutide is independent only of weight loss, acting on the pathophysiology of HFpEF, reducing systemic inflammation (decrease in CRP) and compensating for HF objectively, which is reflected in the decrease in natriuretic peptides (where weight loss alone tends to increase BNP, as seen in studies with patients in the postoperative period of bariatric surgery).^45^ Therefore, HFpEF, in the presence of obesity, seems to have semaglutide as a “wild card” and effective treatment.

## Conclusion

For all these reasons, after years without targeted therapies impacting HFmrEF/HFpEF, two drugs, SGLT2 inhibitors and Finerenone, are reshaping their management. This “Dynamic Duo,” akin to the “Fantastic Four” in HFrEF, is now expected to receive a joint recommendation in clinical guidelines for HF treatment in patients with LVEF >40%. Semaglutide, in turn, seems to provide benefits in this context associated with obesity and may, therefore, be indicated.

Although encouraging, new publications are still needed aiming at treatments that have a definitive impact on mortality. However, in a clinical landscape where previous therapeutic options failed to reduce outcomes, the “Dynamic Duo” marks a turning point in addressing morbidity and mortality in this subset of HF.
